# On Mental Derangement, as it Occurs after Parturition, and During Lactation

**Published:** 1822-09-01

**Authors:** 


*De l'Alienation Mentale des Nouvelles Accouchees et des Nourrices:—that is, On Mental Derangement, as it occurs after Parturition, and during Lactation.* By M. Esquirol, Physician in Ordinary to the Salpetriere. (Annuaire Medico-Chirurgical.)

Many are the maladies to which woman is subjected while performing her allotted function of recruiting the species. Of these a very distressing one is that temporary alienation which so frequently succeeds parturition. It is a charitable, and we trust not a chimerical, supposition of M. Esquirol, that many of those shocking instances of infanticide, which we hear of, are the result rather of momentary derangement of the intellect than deliberate cruelty. Thus he knew a young woman become pregnant, and who did not conceal her pregnancy. She made preparations for her accouchement. She lay-in in the night, and next morning the child was found lying in the privy mutilated by numerous wounds made apparently by scissors. The mother was found in bed. She was conveyed on a litter to some miles distance, and during the journey uttered some incongruous expressions. Being interrogated three days afterwards, she avowed the crime, made no defence, and did not appear to express or feel the slightest regret at the horrible transaction. She refused all kind of sustenance. Is it not evident, says our author, that this unhappy young woman laboured under some aberration of the intellect ?—We are of his opinion.

The number of women who become deranged after parturition and during lactation, is more considerable, M. Esquirol asserts, than is commonly imagined. Of the females received into the Saltpetriere a tenth or twelfth have become insane under the abovementioned circumstances. This affection is much more common in high, than in low life. It is curious, however, that in the *former* class, it seldom takes place after weaning; whereas, it is of comparatively frequent occurrence in the poorer orders of females, after the removal of the child from the breast—a fact which may admit of some physiological speculation, but which our author accounts for by the care which the rich take, and can take, of themselves at such periods.

M. Esquirol refers to the 3d book of the Epidemics of Hippocrates for instances of this disease, and quotes the 14th case as a probable example, in the following words.

> " Peut-etre l'observation XIV. est-elle une manie aigue. Il s'agit de la femme d'epicrate, qui, ayant accouchée de deux jumeaux, delira dès le deuxieme jour de l'accouchement, et mourut phrenetique le vingt et uniéme."

The practice of frequently quoting the works of Hippocrates, and finding descriptions of all diseases in these ancient records, induced us, many years ago, to carefully con over every line of the multifarious writings attributed to the coan sage. The result was not what might be expected. Instead of being impressed with admiration, or rather adoration of these writings, we became thoroughly convinced that they were a chaos of incongruous facts and absurdities which far from repaid the labour of wading through them. We shall probably be deemed Goths or Vandals for this declaration; but it has the merit of being a candid one. To shew how loosely, and sometimes falsely, the " divine old man" is often quoted, we shall here insert the original case, being a short one, which our author has alluded to, as a specimen of puerperal mania two thousand years ago.

"Ægrotus decimus quartus. In Cyzico mulierem, quæ gemellas filias magna difficultate peperit, et non valde purgata est, primum invasit febris horrida, acuta. Capitis et colli gravitas cum dolore. Insomnis ab initio, taciturna ac tetrica et non obsecundans. Urinæ tenues et decolores: siticulosa, anxia plerumque, alvus erroneo modo turbata, et rursus adstricta. *Sexta, ad noctem multum delirabat*, nihil dormivit. Circa undecimam insaniit, et rursus resipuit. Urinæ nigræ, tenues: et rursus tempore interposito oleosæ. Et alvus multis tenuibus turbata. Decima-quarta convulsiones multæ, extremæ partes frigidæ, nihil amplius intelligebat. Urinæ suppressæ sunt. Decima sexta voce destituta est. Decima-septima mortua est: phrenitis." *Popularium, lib.* III. *Sect.* III.

Now we can see nothing in this case but a common instance of puerperal fever, not puerperal mania. She was seized, on what day after delivery is not stated, with ardent fever, and it was not till the sixth day—(not the second, as Esquirol says)—that she became delirious. She died too, on the 17th day, (not the 21st as our author has it,) the disease being pronounced phrenitis by Hippocrates. Thus we see how many errors have crept into a single case, and how little satisfactory is the case itself after all.*

Levret and Zimmerman observe that mania is to be apprehended when the lochia do not flow properly, and when the breasts do not swell by the afflux at the natural period; but experience shows that other affections are the more common consequences of disturbances in the lochial discharge.

---

* Our French brethren, of all other people, pride themselves the most on being strict disciples of Hippocrates—that is to say, of carefully watching diseases, and religiously avoiding all interference with their progress. The wildest absurdities of the coan sage, are as much respected by these lively and ingenious people, as those common truths or facts, which the "divine old man" could not help stumbling on. Thus, our author seems to marvel much that Hippocrates should be wrong in the following aphorism: "Mulieribus quibuscunque ad mammas sanguis colligetur, insaniam significat." *Lib.* V. *Aph.* 40. He quotes several instances of a directly contrary nature. We believe there is hardly a charlatan in London—even the water-doctor in Berners' Street, that would not be ashamed of issuing such an aphorism as that which *lies* in immediate sequence of the one quoted by M. Esquirol. "Mulierem si velis cognoscere, an prægnans sit, ubi dormire volet (incænatæ) aquam mulsam bibendam dato. Et siquidem tormen habuerit circa ventrem, prægnans est: si vero non, prægnans non est." *Aph.* 41, *Lib.* V. Such are the absurd puerilities that command our respect merely because they are enveloped in the rust of antiquity! But why should it be called antiquity? Is not the world older now than it was three thousand years ago? and are not *we* consequently the *antiqui*, both in time and knowledge?

Of 92 cases of puerperal mania, in our author's establishment, sixteen became insane between the first and fourth day of confinement. Twenty-one became alienated between the fifth and fifteenth days—seventeen between the fifteenth and sixtieth days—nineteen between the second and twelfth months of lactation. Nineteen were immediately affected with insanity on weaning their children.* From these facts it would appear—1st, that mental derangement is more frequent among those recently confined, than among those giving suck—2dly, that the danger diminishes in proportion to the length of time that has elapsed since the accouchement—3dly, that women are much more subject to the complaint immediately after weaning, than during lactation.

Puerperal mania is sometimes presaged, even during pregnancy, by melancholy presentiments in the mind; while exaggerated or unfounded apprehensions immediately prelude the explosion of delirium. Sometimes, however, the mania breaks forth without any premonitory symptoms. At the commencement of the disease there is a febrile state of the system present. Yet the skin is soft and moist, the countenance pale, tongue white, breasts flaccid. The abdomen is neither tense nor painful; but sometimes there is acute pain in the uterine region. The pulse is generally small, feeble, and concentrated—mean time there is either exclusive delirium; that is monomania, or aberration of intellect, on a single subject; or, more commonly, general mania. Sometimes profound stupor precedes phrenitis, which may be confounded with puerperal mania, unless attention be paid to the head-ache, redness of the eyes, aridity of the skin, tinnitus aurium, anomalies of the pulse, subsultus tendinum, &c. which attach to the former malady, and distinguish it from the latter. Phrenitis, under puerperal circumstances, is generally mortal about the third or fourth day—rarely passing the seventh—the duration of mania may be prolonged to many months. Those mental alienations which arise during or subsequent to lactation, differ very little from those attendant on parturition, excepting a peculiarity of countenance which can only be known by experience.

The age at which this complaint most frequently occurs is that between twenty-five and thirty years—a period of life most prone to maniacal disorders from whatever cause.

---

* Dr. Gooch, in his excellent paper on puerperal mania, analyzed in the first volume of this series, page 615, does not appear to allude to weaning in the etiology of the disease. Yet the 4th case, p. 620, seems to be one dependent on this cause, and exemplifying M. Esquirol's observations.

*Etiology of Puerperal and Lactation Mania.* Hereditary predisposition—extreme susceptibility—former attacks of the same disease—these predispose to, and even in a few instances excite the malady. What is curious there have been women who became insane *only* when they bore a male child—others who suffered after every *second* accouchement—and some who became alienated regularly in the third or fifth month of suckling.

The *exciting* causes are, for the most part, errors in regimen and moral affections. Of the former class, impressions of cold, however applied, form the most considerable proportion. The sudden weaning of the child, where proper precautions are not taken, becomes a frequent cause of this disease. The moral causes are to the physical, as one to four in proportion. In all ages the influence of mental emotions on parturient females has been duly appreciated. In ancient Rome a crown was suspended over the door where women were confined, to intimate that the house was a sacred asylum for the time. A nearly similar custom exists at Haerlem to this day. The panic of 1814, when the allies entered France, was a prolific cause of puerperal mania. Eleven out of thirteen that entered the Saltpetriere that year, were attributable to this cause. The same happened in 1815, when Napoleon recommenced the scene of warfare and desolation. Causes which, under ordinary circumstances, would be productive of no inconvenience, act most powerfully and often destructively on the parturient female. Thus, 1st, a woman was safely delivered. Next day she sprinkled her bed with odoriferous distilled waters. The lochia are immediately suppressed—no milk comes to the breasts—mania is evinced the same evening, and continues ten months. 2d. A man threw a pail of water over his wife who had lately been confined. She became immediately maniacal, and never recovered from it! 3d. A young woman, 18 years of age, concealed herself and was confined in a granary during very cold weather. She became maniacal, and continued so for twelve months. 4th. A woman giving suck was overtaken by a storm—heated herself by running fast—and in that state crossed a brook knee-deep in water. The milk was suddenly suppressed, and melancholia ensued. 5th. Another woman, during lactation, was frightened by a thunderstorm. The milk disappeared from the breasts, and she instantly lost her reason.

Although a very rare occurrence, yet our author has known a few instances where puerperal mania took place without any suppression of the lochia. The same may be said of the lacteal secretion. Our author enters into some discussion, whether or not there is an actual metastasis of the lacteal secretion to the brain in this disease, and to the peritoneum in puerperal fever. We need hardly say that such a question would not now be agitated on this side of the channel. Metastasis of *action*, not of *matter*, is the prevailing doctrine of British pathologists.

Mental alienation succeeding accouchement is generally curable. Where there was not too strong a predisposition to insanity more than half of our author's patients were cured—that is, out of 92 there were 55 restored to sanity. These restorations were marked by a return of the lochial discharge—by the accession of milk to the breasts—by copious leucorrhœa—by a mucous, sometimes sanguinolent diarrhœa—by a return of the menses suppressed during pregnancy—by subcutaneous abscesses—very rarely by pregnancy.

Of 55 cures, four were within the month—seven within two months—six within three months—seven within four months—five within the fifth month—nine within the sixth month—17 required more than six months—and two required two years and upwards.

Of the whole 92 cases, there were but six deaths. One six months after accouchement—one after a year—two after eighteen months—one after three years—one after five years. How is it, our author asks, that abdominal affections succeeding parturition are so often mortal, while cerebral affections succeeding the same state, are so little so? We cannot give a solution of this problem.

Dissection, M. Esquirol observes, has not disclosed any thing particular in the pathology of puerperal mania. He has sometimes found in these, as in other instances of insanity, albuminous exudations between the meninges; but he asserts, and we implicitly believe him, that he has not found either milk or lochia extravasated within the cranium. We need not inform our readers that nothing has ever been found in the brains of maniacs, that has not been far more frequently found in the brains of people who were not maniacs. Consequently, while we believe that insanity is a corporeal disease, we know not in what the physical lesion or alteration of structure (if indeed alteration of structure be necessarily involved in the complaint) precisely consists. If we find albuminous exudations, turgescence of vessels, or other marks of increased vascularity after death, in the brains of the insane—and if we find all these, times out of number, in the brains of other people who evinced no symptom of insanity, we certainly are not authorized so much by direct proofs as by analogy, to decide on the corporeal seat or cause of this dire affliction.

In respect to treatment, our author properly observes that puerperal and lactation insanity requires the same moral and physical management as mental derangement from other causes. Separation from friends, hygiene, and moral means, are equally necessary, though not always so successful, by themselves, as in other instances of alienation of mind.

Venesection, M. Esquirol observes, should be employed with caution. Leeches to the pudendum or thigh, where there are symptoms of plethora, are more useful. Cupping-glasses, blisters, or sinapisms applied to the thighs, insides of the legs, or nape of the neck, with gently sudorific or laxative diluent drinks, ought, he thinks, to be preferred to more energetic means. Some cases of this disease succeeding parturition were cured by purgative glysters administered twice or thrice a day, the patient at the same time observing an abstemious regimen. Emetics, often repeated, have proved successful also in a few cases. Blisters which, at the beginning or during the period of irritation, did not prove serviceable, were found very useful at a more advanced period of the disease. The warm bath, especially the hip-bath, were admirable auxiliaries to other means.

### CASES.

In conformity with the design of the Annuaire, our author confines himself to those cases which occurred in Salpetriere, though his private practice furnished him with cases of a more interesting nature than this public establishment.

*Case* 1. P. E. ætat. 30, has several relations insane. She was married at the age of 20, and has borne five children. When in the 4th month of pregnancy with her fifth child, she was frightened by a soldier running with a naked sword through the streets. From that time she had a presentiment that something would go wrong in labour, and that she would become insane. She was safely delivered on the 15th April, 1811. Three days afterwards she had uterine hæmorrhage to an alarming extent, which continued for three or four days. Milk now came to the breasts, and she suckled her infant. On the 29th day she became delirious—exhibited extravagancies—and attempted suicide. She was obliged to be tied down to her bed for 15 days, during which she obstinately refused meat and drink. She was conducted to the hospital on the 25th July, being then melancholy, taciturn, the abdomen swelled. The child was removed from the breast. *27th July*. She was furious to-day. Laxative drink—a blister to the nape of the neck—camphorated liniment to the breasts. A leucorrhœal discharge. *20th August*. The blister is allowed to heal—she appears better—eats a little—speaks and walks about of her own accord. In the beginning of October she relapsed, and blisters were again applied, which were followed by a slight febrile movement in the system. On the 20th November she was permitted to see her parents and behaved well. On the 10th December she was restored to her family in perfect sanity."

*Case* II. This was a woman 51 years of age, who entered the hospital, 30th June, 1812. She has a sister, who became insane after an accouchement, and remains deaf since that time. She herself married at 25 years of age. At 26 bore a child, and became insane, the disease continuing till her second pregnancy, when it ceased. The second parturition was not followed by any accident. After this she was eleven times confined, and each accouchement was succeeded by an alienation of a month or six weeks' continuance. At the age of 39 she experienced an attack of apoplexy, followed by hemiplegia. At 47, she had a fever, which was followed by mania that lasted five months. The menses now became irregular, accompanied by head-aches; and they disappeared at 49, without accident. One year after this period, she experienced another attack of fever; and again the menses appeared, and flowed regularly for twelve months. At the age of 51, the death of her husband and other domestic afflictions reproduced mania, and now it was that the patient entered the Saltpetriere. In about six months she was so far recovered as to be able to leave the hospital; but with her nerves so weak that she was liable to be frightened by very trifling causes. We shall quote but one more case out of the ten detailed by our author. It was a fatal one, and the dissection is appended.

*Case* III. " L . . . . . ætat. 41 years, entered the hospital on the 28th November, 1811. At the age of 18 a fright suspended the catamenia, and she became melancholic for eighteen months, after which she resumed her usual health. She was confined in her 36th year, and, on the fourth day after delivery, being alarmed by the midwife quitting her, the milk was suppressed—she refused to eat—became furious—her tongue paralytic. After two months the menses re-appeared—but the patient fell into profound melancholia, and entertained various chimerical and ridiculous notions. When she entered the hospital the malady had continued five years. Her countenance was pallid—features drawn—and she appeared to squint. She refused to eat or lie down in bed for several days. Her head was shaved. On the 5th December, she experienced a stroke of apoplexy; but gradually recruited a little from its effects. In January, a diarrhœa reduced her strength, and she sunk on the 20th of that month.

" *Dissection.* Skull remarkably thick and hard—vessels of the pia mater injected—brain soft—medullary substance injected—pia mater in some places thickened—parietes of the lateral ventricles adherent in some places, with serosity in other parts, and their lining membrane injected, as well as the plexus choroides. Heart enlarged—liver granulated—mucous membrane of the intestines inflamed, and, in some places, sphacelated."

In closing this short analysis we may observe that, although no particular therapeutical indications are broached in this paper, our readers will find it advantageous to bear in mind the information it contains. It is necessary, when we are called to a complaint, especially where it is not a very common one, to be acquainted with the probable issue of the disease—a species of knowledge which can only be acquired from comparisons on a large scale, such as M. Esquirol has here presented. We therefore recommend the paper to the particular attention of our junior brethren.

